# Toward the Discovery of a Novel Class of YAP–TEAD Interaction Inhibitors by Virtual Screening Approach Targeting YAP–TEAD Protein–Protein Interface

**DOI:** 10.3390/cancers10050140

**Published:** 2018-05-08

**Authors:** Floriane Gibault, Mathilde Coevoet, Manon Sturbaut, Amaury Farce, Nicolas Renault, Frédéric Allemand, Jean-François Guichou, Anne-Sophie Drucbert, Catherine Foulon, Romain Magnez, Xavier Thuru, Matthieu Corvaisier, Guillemette Huet, Philippe Chavatte, Patricia Melnyk, Fabrice Bailly, Philippe Cotelle

**Affiliations:** 1 University of Lille, CHU Lille, INSERM-UMR-S 1172-JPArc-Centre de Recherche Jean-Pierre Aubert Neurosciences et Cancer, F-59000 Lille, France; floriane.gibault@ed.univ-lille1.fr (F.G.); mathilde.coevoet@inserm.fr (M.C.); manon.sturbaut@etu.univ-lille2.fr (M.S.); romain.magnez@inserm.fr (R.M.); xavier.thuru@inserm.fr (X.T.); matthieu.corvaisier@inserm.fr (M.C.); guillemette.huet@inserm.fr (G.H.); patricia.melnyk@univ-lille2.fr (P.M.); philippe.cotelle@univ-lille1.fr (P.C.); 2 University of Lille, CHU Lille, INSERM-U995 LIRIC-Lille Inflammation Research International Center, F-59000 Lille, France; amaury.farce-2@univ-lille2.fr (A.F.); nicolas.renault-3@univ-lille2.fr (N.R.); philippe.chavatte@univ-lille2.fr (P.C.); 3 University of Montpellier, CNRS-UMR5048, INSERM-U1054, Centre de Biochimie Structurale, 29 rue de Navacelles, F-34090 Montpellier, France; frederic.allemand@cbs.cnrs.fr (F.A.); guichou@cbs.cnrs.fr (J.-F.G.); 4 University of Lille, CHU Lille, Plate-forme d’Interactions Moléculaires, F-59000 Lille, France; anne-sophie.drucbert@univ-lille2.fr (A.-S.D.); catherine.foulon@univ-lille2.fr (C.F.); 5 ENSCL, F-59000 Lille, France

**Keywords:** protein-protein interaction, YAP-TEAD disruption, molecular docking, binding assays, anticancer

## Abstract

Intrinsically disordered protein YAP (yes-associated protein) interacts with TEADs transcriptional factors family (transcriptional enhancer associated domain) creating three interfaces. Interface 3, between the Ω-loop of YAP and a shallow pocket of TEAD was identified as the most important TEAD zone for YAP-TEAD interaction. Using the first X-ray structure of the hYAP_50–71_-hTEAD1_209–426_ complex (PDB 3KYS) published in 2010, a protein-protein interaction inhibitors-enriched library (175,000 chemical compounds) was screened against this hydrophobic pocket of TEAD. Four different chemical families have been identified and evaluated using biophysical techniques (thermal shift assay, microscale thermophoresis) and in cellulo assays (luciferase activity in transfected HEK293 cells, RTqPCR in MDA-MB231 cells). A first promising hit with micromolar inhibition in the luciferase gene reporter assay was discovered. This hit also decreased mRNA levels of TEAD target genes.

## 1. Introduction

The Hippo pathway regulates cell proliferation, cell death and cell differentiation in multicellular organisms to ensure normal tissue development [1,2,3]. It consists of a complex kinase cascade including successively Merlin, MST1/2 (mammalian ste20-like protein kinase), SAV1 (scaffold protein salvador), LATS1/2 (large tumor suppressor kinase) and MOB proteins (mps one binder kinase) [4]. The final output of Hippo signaling is the inhibition of the transcriptional co-activators YAP (yes-associated protein, Yorkie in Drosophila) and TAZ (transcriptional co-activator with PDZ-binding motif) by the LATS/MOB complex phosphorylation [5]. Phosphorylation of YAP residue S127 (or TAZ S89) induces the binding to the 14-3-3 protein and its subsequent cytoplasmic sequestration and proteasomic degradation [6]. The binding of nucleic TEAD (TEA/ATS domain) transcription factors with VGLL (Vestigial-like) proteins represses the transcription of target genes involved in cell proliferation and apoptotic resistance and leads to the stop of organ growth [7,8]. Conversely, when the Hippo pathway is inactivated, YAP and TAZ translocate into the nucleus, forming complexes with TEAD factors, promoting transcription of target downstream genes such as CTGF (connective tissue growth factor) [9], Cyr61 (cysteine-rich angiogenic protein 61) [10] and AXL [11]. TEAD proteins seem to be the key mediators of the cell growth and the tumorigenic potentials of YAP/TAZ. Deregulation of the Hippo pathway induces the activation or overexpression of YAP/TAZ and/or TEAD. The central role of TEAD and its coactivators [12] such as YAP/TAZ [13,14,15] in many cancers such as lung, thyroid, skin, ovarian, colorectal, prostate, pancreas, esophagus, liver and breast cancer [1,10] has been largely reviewed.

Thus, inhibition of YAP/TEAD interaction is a relevant strategy for cancer therapeutic intervention. Liu-Chittenden Y. et al. were the first to demonstrate the feasibility and relevance of this strategy by identifying verteporfin and related porphyrins as YAP ligands and inhibitors of YAP-dependent transcriptional activity of TEAD [16]. In this context, our team synthesized and tested dipyrrin derivatives, which represent half of the structure of porphyrins [17]. Several groups have developed upstream strategies to induce YAP/TAZ phosphorylation with activators of MST1/LATS1 kinases [18], or LATS1/2 kinases [19,20], and inhibitors of tyrosine kinase Yes-1 [21]. Inhibition of the nuclear localization of YAP/TAZ was also induced by statins via the inhibition of HMG-CoA [22]. Downstream strategies included the discovery or design of compounds competing with TEAD binding [23]. A cyclic peptide derived from YAP_84–100_ sequence [24], a hybrid peptide (named SUPER TDU) derived from VGLL4 protein and YAP_74–99_ sequence [25] and more recently, a cysteine-rich peptide TB1G2 [26] were shown to disrupt the YAP/TEAD interaction. So far, reported non-peptide inhibitors included a family of isothiazole-1,1-dioxides [27], niflumic/flufenamic acids [28] and a family of 3-(alkylthio)-4*H*-1,2,4-triazoles [29]. The modulation of TEAD transcriptional activity with small molecules is of course a challenge [30], but it can be overcome through biological screening of existing chemical libraries. Such a strategy based on biological screening led to the discovery of verteporfin by Liu-Chittenden Y. et al. [16] and a fused tricyclic compound CA3 by Song S. et al. [31]. Another strategy involves a virtual screening of chemical libraries against the TEAD protein, which is possible since the crystal structures of YAP/TAZ-TEAD complexes have been recently solved [32,33]. Then, the selected virtual hits are purchased or synthesized for subsequent testing.

Herein, we report the virtual screening of a subset of compounds from ZINC database, the selection of four virtual hits fitting into a specific zone of the YAP-TEAD interface and their biophysical and biological evaluations.

## 2. Results

### 2.1. X-Ray Crystallographic Structure Analysis and Virtual Screening

**Figure 1:**
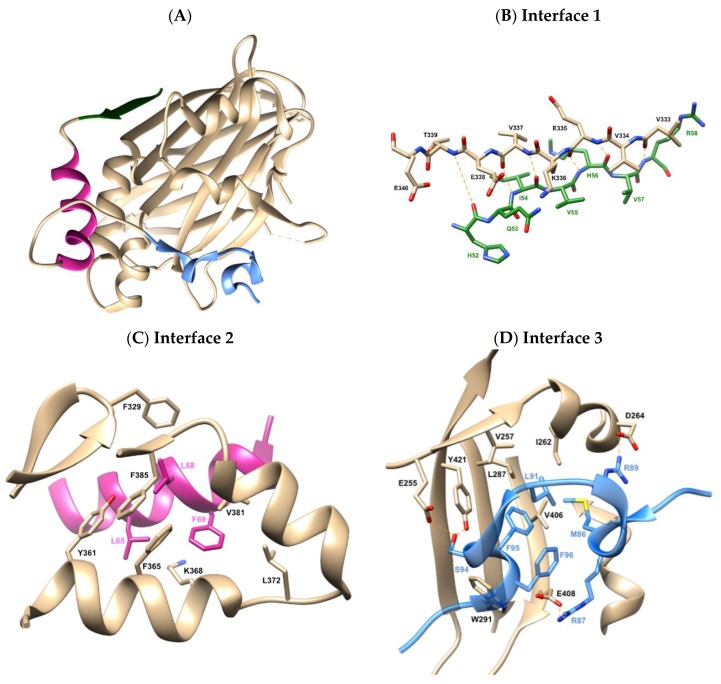
Structure of the hYAP_50–171_-hTEAD1_209–426_ complex (PDB 3KYS) (**A**) [32] with three interfaces between hYAP_50-171_ in green (**B**), cyan (**C**) and blue (**D**) and hTEAD1_209-426_ (in light brown).

Before performing the virtual screening, careful examination of the X-ray structures of YAP/TEAD complexes was necessary. At the beginning of the project, only two structures mYAP_35–92_-mTEAD4_210–427_ (PDB 3JUA) [33] and hYAP_50–171_-hTEAD1_209–426_ (PDB 3KYS) [32] were readily available. We took advantage of the last one, which shows that the TEAD-binding domain of YAP wraps around the globular structure of TEAD via three interfaces (Figure 1). Interface 1 is mediated by seven intermolecular hydrogen bonds between the peptide backbone of YAP β1 sheet (residues 52–58) and TEAD β7 sheet (residues 333–339) forming an antiparallel β sheet.

The second interface involves the YAP α1 helix (residues 61–73) and TEAD α3 and α4 helices (residues 360–384). Interactions are mainly driven by hydrophobic contacts between L65 of YAP and Y361, F380, K368 of TEAD, L68 of YAP and F329, F365 and F385, L69 of YAP and F365, K368, L372, V381 of TEAD. In the third interface, the twisted-coil region (named Ω) of YAP (residues 86–100) interacts with TEAD by fitting side chains into the deep pocket, which is formed by β4, β11, β12, α1 and α4 of TEAD. Particularly, the hydrophobic side chains M86, L91, and F95 of YAP make multiple van der Waals contacts with I262, V257, L287, V406, and Y421 of TEAD. The interaction is further strengthened by interactions between the guanidinium group of YAP R89 and the carboxylate oxygen of TEAD D264, and two hydrogen bonds formed by the side chain of YAP S94: one with Y421, and the other with E255 of TEAD. Several complementary studies have shown that the Ω loop (85–99 sequence) is essential for efficient binding with TEAD [30,34,35]. On the other hand, mutation studies on TEAD indicated that only the Y421A and Y421H mutations strongly reduced the interaction with YAP [28]. Since Y421 is located in interface 3, these results also support the critical role of interface 3 in the YAP/TEAD complex formation. For the most efficient strategy of YAP/TEAD disruption, we found it more rational to target TEAD than YAP since the nuclear TEAD acts as a downstream effector of the Hippo pathway.

**Scheme 1:**
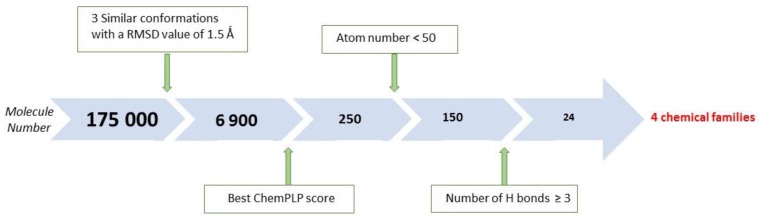
Virtual screening method.

**Figure 2:**
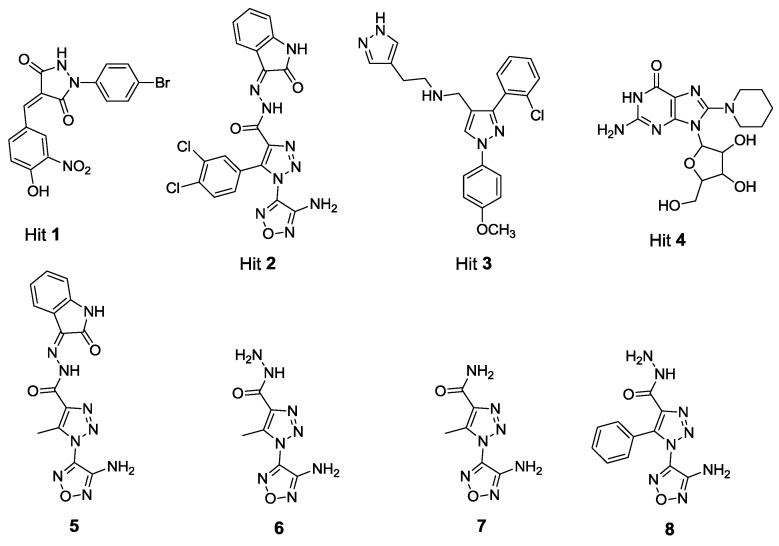
Structure of the four selected virtual hits and some related derivatives.

**Figure 3:**
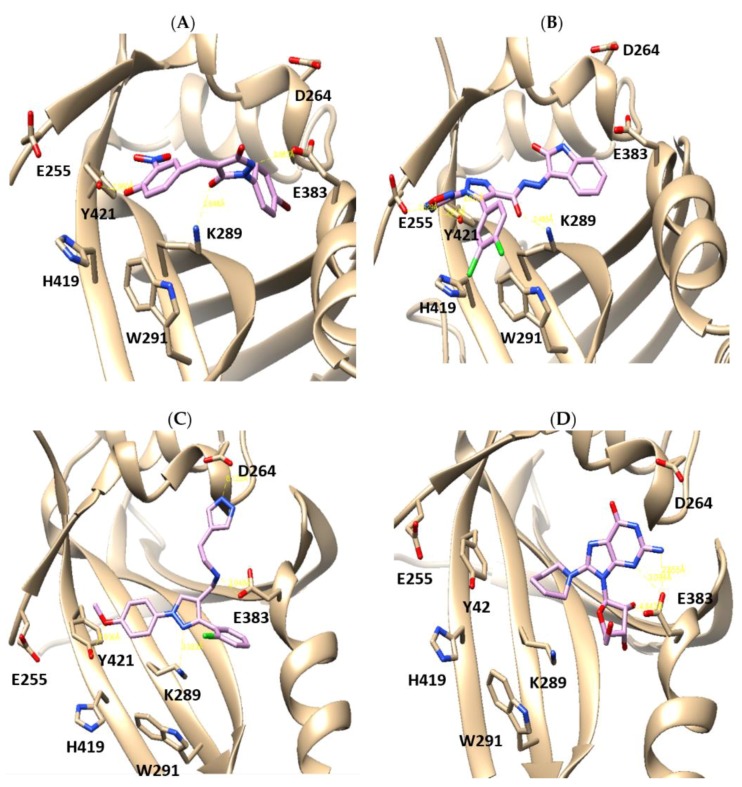
Docking pose in the hTEAD1_209-426_ binding pocket (interface 3) of each virtual hit (hit **1**), (**A**); hit **2**, (**B**); hit **3**, (**C**); hit **4**, (**D**) using the UCSF Chimera software.

Our virtual screening (Scheme 1) was based on hTEAD1_209–426_ structure and molecules were docked into the binding cavity formed by the C-terminal hTEAD1 region contributing to interface 3. Moreover, all TEAD isoforms display a high sequence homology in the C-terminal domain [30], making the simultaneous targeting of all TEAD isoforms possible. We used the ZINC database, a freely available database of commercially available compounds in ready-to-dock 3D formats. First, filters were applied to the compounds to exclude any potential toxic ability and to enrich the library with the most relevant protein–protein interaction (PPI) inhibitors [36,37]. A molecular weight of >400 Da was applied to the ZINC database, followed by a previously developed Bayesian model on the basis of 42 topological descriptors and 2D fingerprints. This led to the selection of a subset of the ZINC database of 750,000 compounds. Amongst them, the first 175,000 were used for an initial screening run. The binding site was defined as a sphere centered on residue K289 at interface 3. Its radius was set to 12 Å in order to include specific residues identified by directed mutagenesis. Ligands were submitted to 100,000 docking operations with a maximum number of ten final poses and with an early calculation termination if three poses were generated within a RMSD (root-mean-square deviation) of 1.5 Å. This constraint led to a decrease in candidate compounds to 6900. Next, the best ChemPLP scored pose was retained for each compound, giving a set of 250 compounds. A score per atom was also calculated by dividing this score by the number of heavy atoms in the compound. The 150 remaining compounds were then ranked according to their propensity to establish hydrogen bonds with the residues of the TEAD binding site and 24 compounds with a number of H-bond donors/acceptors ≥3 were retained. Finally, an inspection of the structures led to four candidates. They were retained for their structural diversity, chemical feasibility and also their apparent best fitting into the binding cavity. Figure 2 and Figure 3 show the structures of the four virtual hits and their docking poses in the TEAD binding pocket using the UCSF Chimera software, respectively.

Compound **1** is a pyrazolidine-3,5-dione substituted by bromophenyl and benzylidene moieties, which fits into the binding pocket forming three hydrogen bonds, one between the heterocyclic nitrogen atom and the carboxylate function of E383 at the entry of the pocket, another one between the opposite carbonyl and the amine function of K289 and a third one between the phenol and the Y421 on the outside of the pocket. The bromophenyl ring fits into the bottom of the pocket, in a position close to that of YAP F95 phenyl side chain. Compound **2** has a more extended structure adopting a strikingly close binding mode, with its indolinone ring in the same position as the bromophenyl ring of compound **1**, albeit slightly less deep. There is a double pronged hydrogen bond between the carbonyl function and the imino nitrogen atom of the hydrazide linker with the amino group of K289. The amine of the oxadiazole interacts with the carboxylate group of E255 and the phenol of Y421. A weaker interaction could come from a stacking between the dichlorophenyl ring and the side chain heterocyclic rings of H419 and W291, but this would require a slight reorientation of these side chains. Compound **3** is embedded deeper in the TEAD pocket than **1** and **2** and wraps E383 with its chlorophenyl ring and its aminoethylpyrazole chain. The secondary amino group in the linker between both pyrazole rings makes an ionic bond with the carboxylate group of E383, a nitrogen atom from the external pyrazole makes a hydrogen bond with the carboxylate function of D264 and the central pyrazole ring makes a hydrogen bond with the amino group of K289. Lastly, the methoxy group could also make a hydrogen bond with the phenol function of Y421, after a conformation change of this residue. Compound **4** is a guanosine substituted by a piperidine ring. Interactions with the binding pocket are mediated by several hydrogen bonds between its sugar moiety, its purinergic primary amino group and E383 (main and side chains). The piperidine ring located in the place of YAP F95 sidechain is not essential for the binding affinity but contributes to the global positioning of the whole molecule in the pocket.

### 2.2. In Vitro and in Cellulo Binding Assays

Once the virtual hits were selected, they were purchased for testing. Firstly, we measured the thermal stability of wt hTEAD2_217–447_ produced under our experimental conditions (see Materials and Methods) using a fluorescence-based thermal shift assay (TSA). In this assay, the melting temperature (*T_m_*) measured for hTEAD2_217–447_ was 51.3 ± 0.3 °C. In the presence of potential ligands, the thermal stability of the protein was changed and positive thermal shifts (Δ*T_m_* = *T_m_* (protein + ligand) − *T_m_* (protein alone)) were measured. Table 1 reports the Δ*T_m_* values for each virtual hit and niflumic acid. This last compound was discovered as the best hit of a series of fenamic acid derivatives. It was shown to bind to hTEAD2_217–447_ (*Kd* = 28 μM) in two distinct binding sites: the palmitate pocket and the interface 3 region [28]. Its binding affinity to TEAD2 is mainly driven by its interaction with the central pocket of TEAD2 rather than the interface 3 region. In this study, niflumic acid should be considered as a comparative TEAD2 binder rather than a reference. So far, no non-peptidic compound interacting specifically with the interface 3 region was firmly described. Table 1 reports the Δ*T_m_* values obtained for each virtual hit and niflumic acid.

Except for hit **4**, the three other hits stabilized hTEAD2_217–447_ protein with Δ*T_m_* values around 3.5–4.0 °C, comparable to that of niflumic acid (Supplementary Figure S1). In our hands, niflumic acid (Δ*T_m_* = 3.5 °C) displayed a better affinity toward hTEAD2_217–447_ than that reported by Pobbati A. V. et al. (Δ*T_m_* = 0.6–1.8 °C) at the same protein concentration [28]. This slight difference might be related to the compound concentration, which in our case is at least two-fold higher (50–250 μM) than that used by Pobbati A. V. et al. (20 μM). Thermal shift data were also recently published by Mesrouze Y. et al. which measured a positive thermal shift of 7.4 °C induced by the binding of hYAP_51–99_ (20 μM) to hTEAD4_217–434_ (1–2 μM) [38]. Thus hTEAD4_217–434_ was better stabilized by hYAP_51–99_ (a shortened fragment of YAP_50–171_, showing the same binding affinity as the whole TEAD-binding region of YAP [32]) than hTEAD2_217–447_ by our molecules. This undoubtedly reveals a lower affinity of our hits. These first results led us to obtain more quantitative data about the interaction between our hits and the hTEAD protein and, herein, we report for the first time a study using microscale thermophoresis (MST) on GFP-labeled hTEAD2_217–447_ in CHO-K1 cell lysate. So far ITC or SPR experiments performed to investigate the interaction of potential TEAD binders with TEAD protein were done on isolated purified TEAD proteins (or TEAD fragments). In presence of GFP-labeled hTEAD2_217–447_ in CHO-K1 cell lysate, the measured *K_d_* values of our hits will be closer to those occurring in physiological conditions.

**Figure 4:**
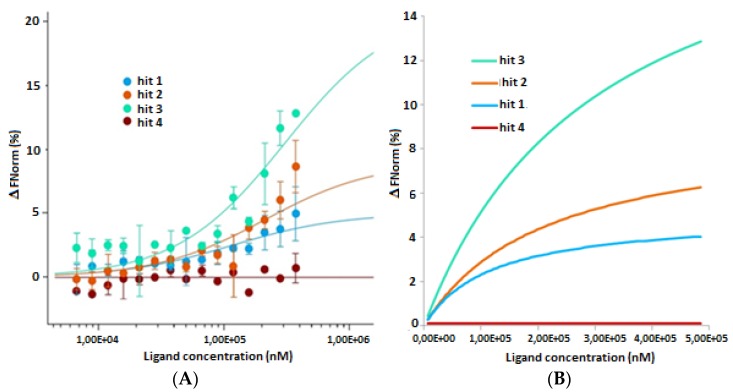
Titration of eGFP-hTEAD2_217–447_ (40 nM) by compounds **1**–**4** in CHO-K1 cell lysate; LED intensity: 100%; MST power: 40%. Fitting of the binding curves by the *K_d_* model (**A**), and a linear *K_d_* model (**B**).

To validate our MST method, the binding of hYAP_50–102_ to GFP-labeled hTEAD4_217–447_ was investigated, yielding a *K_d_* value of 96 nM in accordance with previous literature data (Supplementary Figure S2) [35]. Then, a first run using high concentrations of compound (200 μM) was done. There was no significant variation of the normalized fluorescence in presence of compound **4** and niflumic acid, in contrast to compounds **1**, **2** and **3**. Compound **4** did not yield any binding curve and so was selected as a negative control. No saturation was observed due to the solubility limit that has been reached for every compound. Nevertheless the signal to noise ratio (>30) was strong enough to conclude that TEAD binding was occurring. Figure 4 shows the binding curves obtained for compounds **1**–**3**.

**Figure 5:**
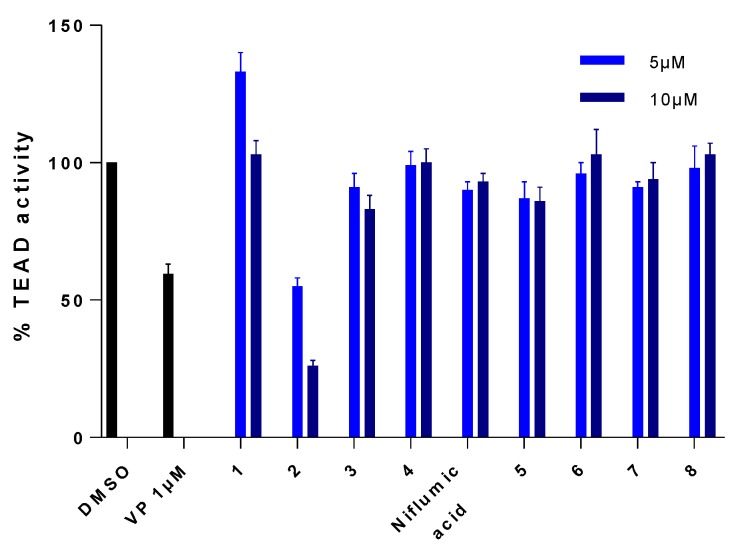
TEAD reporter luciferase activity observed in HEK293T cells treated with 5 and 10 μM hit compounds **1**–**4**, niflumic acid and verteporfin (VP, 1.0 μM) after 24 h post transfection.

**Figure 6:**
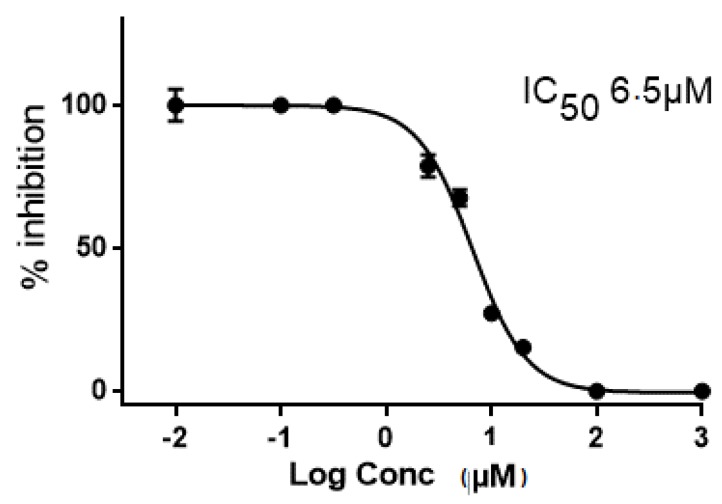
TEAD reporter luciferase activity inhibition observed in HEK293T cells treated with compound **2** after 24 h post transfection.

Fitting of the binding curves by a linear *K_d_* model (Figure 4B) led to a better appreciation of the saturation phase for hits **1** and **2** than by the *K_d_* model (Figure 4A). *K_d_* values above 300 μM for compounds **1**–**3** (392, 650 and 363 μM, respectively) were determined. We next inquired if the in vitro TEAD binding of these three hits revealed by TSA and MST tests would have biological consequences in cellular assays. Firstly, we measured the TEAD transcriptional activity in transfected HEK293T cells in the presence of our compounds using a TEAD reporter construct. A luciferase reporter is placed downstream of TEAD sites; therefore, the expression level of luciferase correlates with TEAD transcriptional activity. We observed a significant decrease in luciferase expression levels in the presence of compounds **2** and **3** (Figure 5; Table 1) at 5 and 10 μM concentrations. The most important decrease was observed with compound **2** (10 μM) with a residual luciferase activity of 36%. At the same concentration, residual luciferase activities were 80% and 86% for compound **3** and niflumic acid, respectively. Interestingly, compound **2** emerged as the most potent compound in the luciferase assay, with an IC_50_ value of 6.5 μM (Figure 6), followed by compound **3** and niflumic acid. Compound **2** at a 5.0 μM dose inhibited to 60% the TEAD transcriptional activity in a manner similar to verteporfin at 1.0 μM (Figure 5).

Altogether, compound **2** appears to be the most effective compound for in vitro and in cellulo YAP-TEAD disruption. In parallel, we purchased analogues of **2**, (compounds **5**–**8**, Figure 2) to try and establish some preliminary structure-activity relationships. Thermal shift measurements showed that only compound **5** retained a noticeable but lowered affinity toward hTEAD2_217–447_ with a two-fold lower Δ*T_m_* value of 1.5 °C (Table 1). For the other derivatives, there was a complete loss of interaction with hTEAD2_217–447_. This was confirmed by the luciferase reporter assay (Table 1) as compound **5** was the only one to induce a moderate inhibition of luciferase activity (14%) comparable to that of niflumic acid. Binding affinity was severely impaired for compounds **6**–**8** lacking the isatin cycle showing the crucial role of this motif for TEAD binding. Comparison of binding parameters obtained for **5** and **8** also revealed the importance of the aromatic phenyl ring at position 5 of the central triazole scaffold. This is particularly reflected in the luciferase reporter assay, where compound **8** induced a 4.5-fold lower inhibition of luciferase activity than **5**.

**Figure 7:**
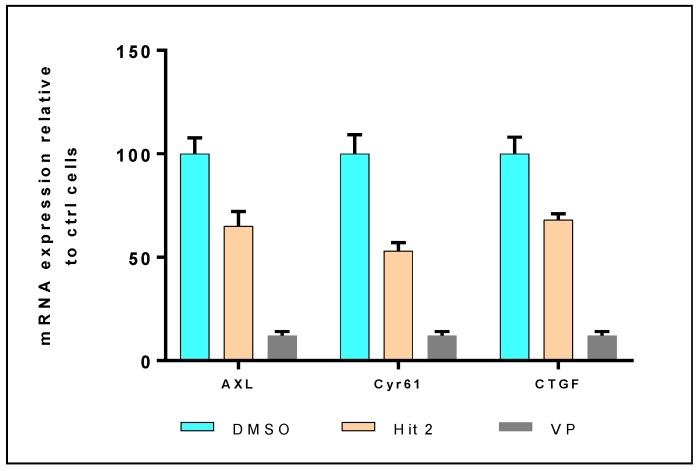
Expression of YAP/TEAD target genes (AXL, Cyr61 and CTGF) in MDA-MB231 cells incubated in the presence of drug (10 μM) for 48 h. mRNAs were measured by RTqPCR. Data are representative of three independent experiments.

Finally we focused on the effect of compound **2** on YAP-TEAD target genes. We selected MDA-MB-231 breast cancer cell line because it similarly expresses YAP and TAZ co-activators, presents a high TEAD transcriptional activity [21] and yields a high expression of gene targets (AXL, Cyr61 and CTGF) [21,22]. We analyzed (by RT-qPCR) the expression of mRNAs of the downstream gene targets AXL, Cyr61 and CTGF (Figure 7). Compound **2** induced a decrease of 35, 47 and 32% for AXL, Cyr61 and CTGF mRNAs expression, respectively whereas verteporfin (VP) was more efficient with 88% inhibition for the three gene levels.

## 3. Materials and Methods

### 3.1. Molecular Modelling

Molecular studies were performed using Gold suite v5.2203 within the Hermes v1.6 GUI (CCDC©). The docking was realized on the TEAD crystal structure from the hTEAD1_194–411_-hYAP1_50–171_ complex (PDB 3KYS). The binding site was defined as a sphere centered on residue K289 at the interface 3. This corresponds to a region where residue F95 of YAP α2 helix fits. Its radius was set to 12 Å in order to include specific residues identified by directed mutagenesis. A subset of the ZINC database (750,000 compounds) was selected in a previous work at the laboratory for their druglikeliness (positive Lipinski’s parameters and known toxic or reactive structures filtered out) and Protein-Protein Interaction inhibition plausibility. The first 175,000 structures from this subset of ZINC database were docked set to 100,000 operations per compound and a maximum of 10 final poses, but with an early termination stopping the calculations if 3 poses were generated within a RMSD of 1.5 Å. The screening was run in parallel on a SGI Altix workstation consisting of 96 titanium 2 processors. Second, the best ChemPLP score pose only was conserved for each compound. A score per atom was also calculated by dividing this score by the number of heavy atoms of the compound, to keep the embedment of the molecule as a guideline. The more buried in the cavity a compound is, the higher its average per atom score. The score per atom reduces the size contribution of a compound to its overall score while keeping higher scores for compounds with a larger number of interactions with the protein. As the number of hydrogen and ionic interactions was more or less conserved for the best scored compounds, the average score per atom is therefore linked to the compound having a larger contact surface with the protein, i.e., being positioned more in depth in the binding pocket. Thirdly, the number of hydrogen bonds formed with the residues of the binding site was extracted and the number of individual engaged residues was also noted in order to differentiate between a possible artefactual multibonding with a single residue and a multiresidue bonding. Lastly, a rough structural clustering of the compounds was realized to increase the immediate accessibility of a large chemical diversity, which would otherwise be invisible in the whole set of solutions.

### 3.2. Chemicals

All chemicals were purchased from Ambinter c/o Greenpharma (Orléans, France) or Sigma-Aldrich (Saint Quentin Fallavier, France) and used without purification. 

### 3.3. Binding Tests

#### 3.3.1. Thermal Shift Assay

The cDNA coding the human TEAD2 sequence (residue 217 to 447) [28] was ordered to Integrated DNA Technologies. This target protein was cloned in frame into a pDBHis-MBP between NdeI and XhoI sites to give the pDBHis-MBP-TEAD2 plasmid. In this construct TEAD2 is fused with a (his)_6_-MBP N-terminal tag. This N-terminal tag is followed by the HRV 3C (3C) protease recognition site (Leu-Glu-Val-Leu-Phe-Gln/Gly-Pro). Specific cleavage occurs between Gln and Gly, with Gly-Pro remaining at TEAD2 protein N terminus. The pDBHIs-MBP-TEAD2 plasmid was transformed in *Escherichia coli* BL21λDE3 cells and grown on LB agar plate with 50-μg/mL kanamycin at 37 °C overnight. Transformed cells were grown overnight at 37 °C in LB media, supplemented with 50 μg/mL kanamycin, until 0.6 OD_280_, then the culture temperature was decreased to 16 °C and production was induced with 0.2 mM of IPTG overnight. Cells were harvested by 20 min centrifugation at 6000× *g* at 4 °C. The pellet was resuspended in 20 mM Tris-HCl pH 7.5, 600 mM NaCl and 2 mM 2-mercaptoethanol (buffer A) and stored at −80 °C. Cells were supplemented with a *Complete^®^* EDTA free tablet (Roche, Meylan, France) lysed by sonication, insoluble proteins and cell debris were sedimented by centrifugation at 40,000× *g* at 4 °C for 30 min. Supernatant was supplemented with imidazole to a 10 mM final concentration, filtered through 0.45-μm filters and loaded onto an affinity column (1 mL His Trap FF, Dutscher, Brumath, France), equilibrated with buffer B (buffer A + 10 mM imidazole). Columns were washed with 20 column volumes of buffer B and proteins eluted with a linear 0–100% gradient of buffer C (buffer A + 300 mM imidazole). The peak fractions were analysed by SDS-PAGE. Fractions containing tagged TEAD2-MBP were pooled. These fractions were dialysed overnight at 4 °C against a conservation buffer containing 20 mM Tris pH 7.5, 600 mM NaCl, 2 mM DTT (Buffer D) and at the same time the (his)_6_-MBP tag was separed from TEAD2 by 3C cleavage (ratio proteine/protease 1000/1 *w*/*w*). Uncleaved protein, (his)_6_-MBP tag and 3C protease were removed by binding onto gravity column containing 2.5 mL Ni-sepharose and 0.5 mL Glutathione sepharose (3C protease being GST-Tagged). Purified TEAD2 did not bind this colunm and was directly collected in the flow through. Purified protein was analysed by SDS-PAGE, concentrated and injected onto gel filtration column (Superdex 75 Hiload 16/60 GE Lifescience) equilibrated with buffer E (20 mM Tris pH 7.5, 100 mM NaCl, 2 mM MgCl_2_, 2 mM TCEP and 5% glycerol). The peak fractions were analysed by SDS-PAGE and the fractions containing pure TEAD2 were pooled.

Thermal shift assay was performed on anABI Prism 7900 HT, originally designed for real-time quantitative PCR, in a 96-well plate format. The gradient method contained 70 cycles and started at 25 °C by rating the temperature of 1 °C/cycle. All experiments were conducted with the hTEAD2_217-447_ protein (20 μM) in the purification buffer [20 mM TRIS (pH 8.0), 100 mM NaCl, 2 mM MgCl_2_, 1 mM TCEP (tris(2-carboxyethyl)phosphine), 5% glycerol]. A dilution of the 5000x SYPRO^®^ Orange commercial solution was first performed in the buffer to obtain a 10x SYPRO^®^ Orange intermediate solution, which was diluted with hTEAD2_217–447_ to obtain a 1–5 μM final protein concentration range. The total volume was kept constant (40 μL) to obtain samples containing constant concentration of SYPRO^®^ orange and varied analyte/protein concentrations. Analyte solutions were prepared from stock solutions at 10 mM in DMSO (dimethylsulfoxide) and added to the mixture to reach a final DMSO concentration of 2.5% and a 50–250 μM compound concentration range. To determine the melting temperature Tm for the protein, a Boltzmann model was used to fit protein unfolding curves using the GraphPad Prism^®^ (v5.02) software.

#### 3.3.2. Microscale Thermophoresis

hYAP_50–102_ was purchased from Xprochem^®^. hTEAD2_217–447_ was cloned into the peGFP-C1 expression vector and synthesized by ProteoGenix SAS yielding a N-terminal green fluorescent protein. CHO-K1 cells (ATCC^®^ CCL-61™) were transfected with the Cell Line Nucleofector^®^ Kit T, Program U-023. After three weeks of selective pressure, the population of cells strongly expressing eGFP-hTEAD_217–447_ proteins was analysed using flow cytometry and retained for microscale thermophoresis analysis.

Cells overexpressing eGFP-hTEAD_217–447_ were suspended in 300 μL RIPA buffer supplemented with a protease inhibitor cocktail. The lysate was collected by centrifugation at 15,000 rpm at 4 °C for 45 min. The whole process is intended to be purification-free [39].

Experiments were performed on a NanoTemper^®^ Monolith NT.115 instrument with blue filters. For each compound, screening with constant hTEAD2 concentrations (10 nM) and 200 μM of ligands was performed in a PBS buffer supplemented with 10% DMSO. Positive control (YAP_50–102_) and buffer reference (PBS 1X; 10% DMSO) were used to identify high and low affinity binders. All measurements were performed at 22 °C. Data analysis was carried out using NTanalysis.

### 3.4. Biology

#### 3.4.1. Cell Cultures

HEK293T (kidney) cell line was purchased from the American Type Culture Collection and cultivated in DMEM media containing 10% of heat inactivated FBS, l-glutamine and penicillin/streptomycin.

CHO-K1 cells (ATCC^®^ CCL-61) were cultured in Ham’s F-12 Nutrient Mixture (F12, LifeTechnologies) with 1% PS (Penicillin-Streptomycin, Life Technologies, Illkirch, France) and 10% FBS (Foetal Bovine Serum, LifeTechnologies). Cells were passaged every 3 days upon reaching confluence, and the absence of mycoplasma contamination was verified (MycoAlertTM Detection Kit, Lonza, Basel, Switzerland). Cell lines were cultured at 37 °C in a 5% CO_2_ atmosphere.

MDA-MB-231 cell line was purchased from the American Type Culture Collection and cultivated in DMEM media containing 10% of heat inactivated FBS, l-glutamine and penicillin/streptomycin.

#### 3.4.2. Luciferase Reporter Assay

HEK293T cells were seeded at a density of 1 × 10^5^ cells in 24 well plates coated with polyethylenimine (10 μg/mL). Cells were transfected with the TEAD luciferase reporter plasmid 8XGTIIC-Luciferase (Addgene reference 34615) and a control β-galactosidase plasmid CMV-βGal using the lipofectamine 2000 Reagent (Life Technologies, Inc.) according to the manufacturer’s instructions. Compounds were tested at different concentrations ranging from 0.25 to 20 μM. After 24 h post transfection, cells were lysed in Reporter Lysis Buffer (Promega, Charbonnières-les-Bains, France) and luciferase activity was measured on the Mithras LB940 plate reader and normalized to β-galactosidase. β-galactosidase control allowed us to qualitatively estimate the cytotoxicity of the tested compounds.

#### 3.4.3. mRNA Expression

mRNAs were extracted from cultured cells or human tumors with the NucleoSpin kit (Macherey-Nagel, Hoerdt, France). Retrotranscription was done on 1 μg of mRNA accordingly to the Advantage RT-for-PCR Kit protocol (Clontech, Saint-Quentin-en-Yvelines, France). PCR was performed using SsoFastTM Evagreen Supermix kit following the manufacturer’s protocol using the CFX96 real time PCR system (Bio-Rad, Marnes-la-Coquette, France). To monitor any change in mRNA expression, we used the ΔΔCt method between one condition and a control condition after normalization with the housekeeper gene RPLP0. Each sample was done in triplicate.

## 4. Conclusions

Starting from the virtual screening of a subset of the ZINC database, we selected four virtual hits fitting into a specific essential zone of the YAP/TAZ–TEAD interface. Biophysical and biological evaluations led to the identification of a promising compound with a micromolar IC_50_ value in a luciferase gene reporter assay, which inhibited mRNA expression of YAP–TEAD target genes. This hit will be the starting point for further chemical explorations aimed at improving its in vitro and in cellulo disruptive potency of the YAP/TAZ–TEAD complex. These studies will be reported in due course.

## Figures and Tables

**Table 1:** Interaction between niflumic acid, the different commercial hits and derivatives and hTEAD2_217–447_ assessed by thermal shift assay ^a^ and microscale thermophoresis ^b^. TEAD reporter luciferase activity in HEK293T cells treated with 10 μM compound after 24 h post transfection ^c^.

Compound	Δ*T_m_* (°C) ^a^	*K_d_* (μM) ^b^	% Luciferase Activity ^c^
**1**	4.2	392	103 ± 5
**2**	3.4	650	36 ± 2
**3**	4.1	363	80 ± 5
**4**	0.6	n. d.	100 ± 5
Niflumic acid	3.5	n. d.	86 ± 3
**5**	1.5	n. d.	86 ± 5
**6**	0.1	n. d.	103 ± 9
**7**	0.1	n. d.	94 ± 6
**8**	0.3	n. d.	103 ± 4

## Supplementary Materials

The following are available online at http://www.mdpi.com/2072-6694/10/5/140/s1. Figure S1: Representative normalized graphs showing a shift in thermal stability of hTEAD2_217–447_ in presence of the potential TEAD2 binders **1**–**4** and niflumic acid. Apo refers to the melting curve of the protein alone. Figure S2: Dose-response curve for the binding interaction between hTEAD2_217–447_ and YAP. The concentration of hTEAD2_217–447_ is kept constant at 10 nM while the YAP_50–102_ concentration varies from 10 µM to 4.8 nM.
