# Supplementary material for: Toward the Discovery of a Novel Class of YAP–TEAD Interaction Inhibitors by Virtual Screening Approach Targeting YAP–TEAD Protein–Protein Interface
Source: Cancers (Basel). 2018 May 8;10(5):140. doi: 10.3390/cancers10050140 (PMC5977113; doi:10.3390/cancers10050140)
Supplement: Supplementary file 1 [file cancers-10-00140-s001.pdf]

Toward the Discovery of a Novel Class of YAP–TEAD Interaction Inhibitors by Virtual Screening Approach Targeting YAP–TEAD Protein–Protein Interface

Floriane Gibault, Mathilde Coevoet, Manon Sturbaut, Amaury Farce, Nicolas Renault, Frédéric Allemand, Jean-François Guichou, Anne-Sophie Drucbert, Catherine Foulon, Romain Magnez, Xavier Thuru, Matthieu Corvaisier, Guillemette Huet, Philippe Chavatte, Patricia Melnyk, Fabrice Bailly and Philippe Cotelle

Supplementary Material

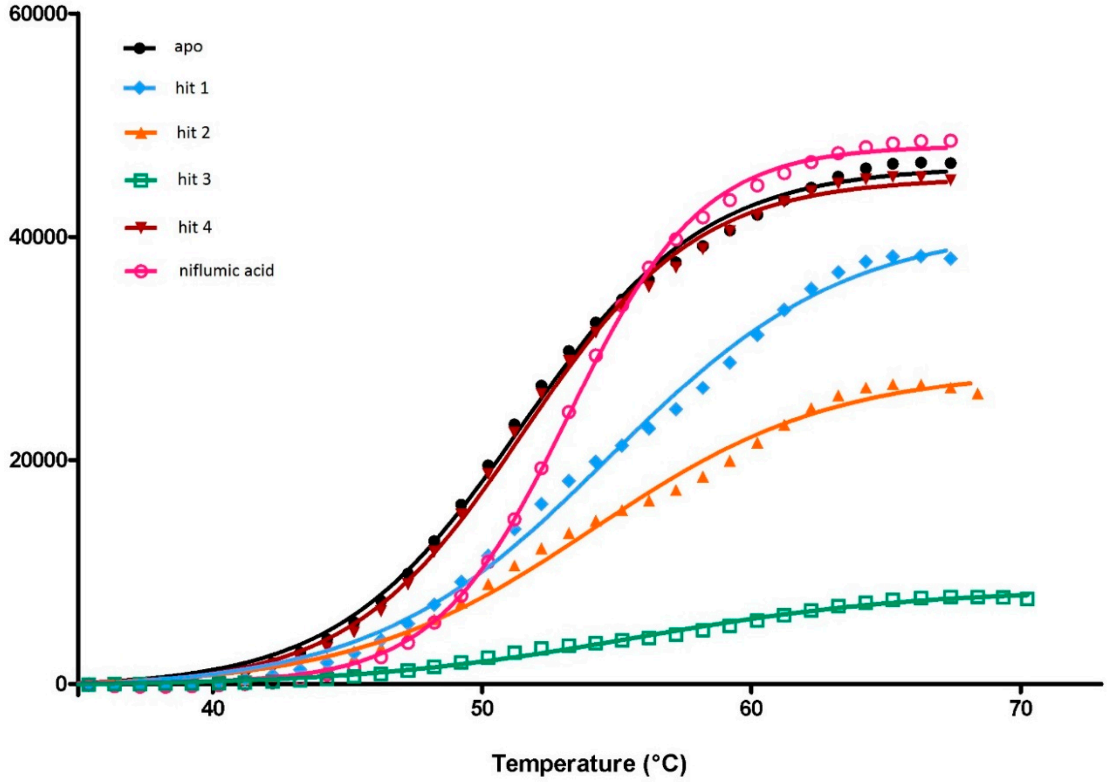

Figure S1. Representative normalized graphs showing a shift in thermal stability of hTEAD2$_{217–447}$ in presence of the potential TEAD2 binders **1-4** and niflumic acid. Apo refers to the melting curve of the protein alone.

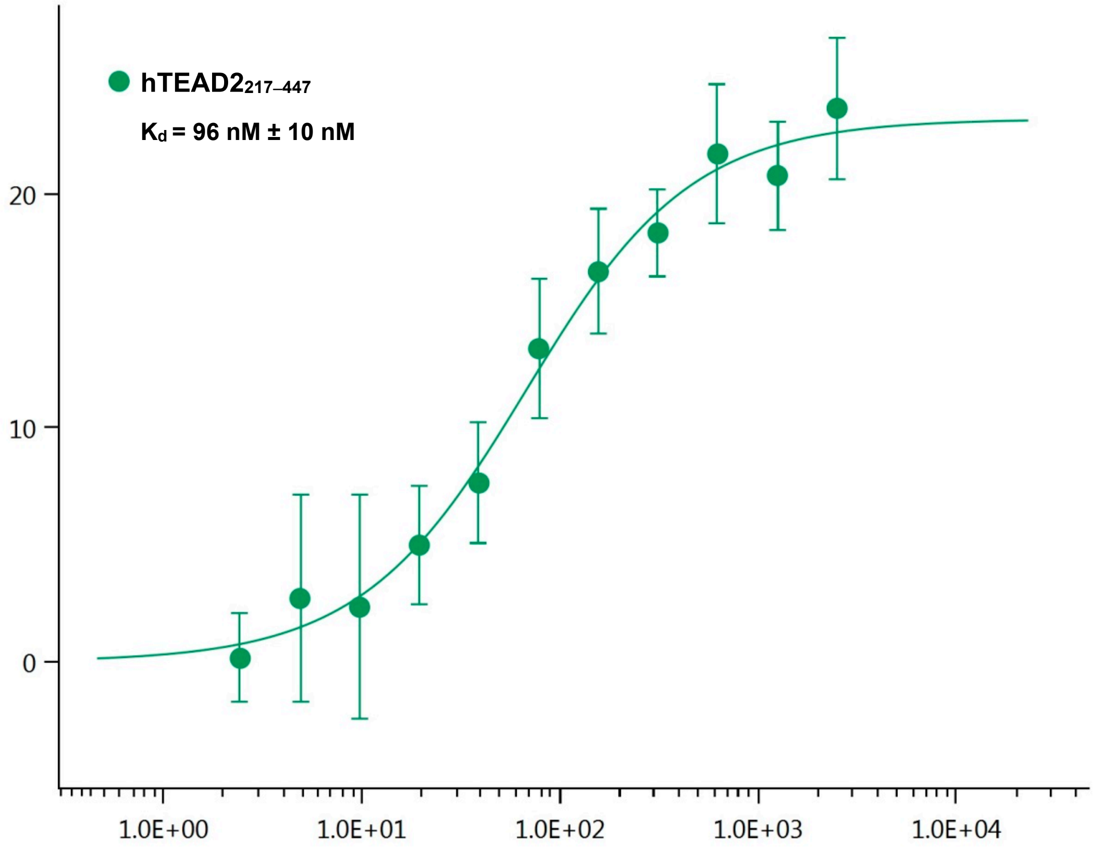

Figure S2. Dose-response curve for the binding interaction between hTEAD2$_{217–447}$ and YAP. The concentration of hTEAD2$_{217–447}$ is kept constant at 10 nM while the YAP$_{50–102}$ concentration varies from 10 μM to 4.8 nM.
